# Supplementary material for: Distinct T-cell receptor (TCR) gene segment usage and MHC-restriction between foetal and adult thymus
Source: eLife. 2024 Dec 5;13:RP93493. doi: 10.7554/eLife.93493 (PMC11620746; doi:10.7554/eLife.93493)
Supplement: Supplementary file 1. [file elife-93493-supp1.docx]

Supplementary File 1

**Supplementary File 1 Antibody panel for staining thymus from 4 week mice.**

| **Antibody** | **Clone** | **Supplier** | **Catalogue number** |
| --- | --- | --- | --- |
| PE anti-mouse CD3 | 17AD | BioLegend | 100206 |
| PerCP/Cyanine5.5 anti-mouse CD4 | RM4-4 | BioLegend | 116012 |
| FITC anti-mouse CD8a | 53-6.7 | BioLegend | 100706 |
