# Supplementary material for: Distinct T-cell receptor (TCR) gene segment usage and MHC-restriction between foetal and adult thymus
Source: eLife. 2024 Dec 5;13:RP93493. doi: 10.7554/eLife.93493 (PMC11620746; doi:10.7554/eLife.93493)
Supplement: Supplementary file 2. [file elife-93493-supp2.docx]

**Supplementary File2 Antibody panel for staining thymus from E18.5 embryos.**

| **Antibody** | **Clone** | **Supplier** | **Catalogue number** |
| --- | --- | --- | --- |
| Brilliant Violet 421 anti-mouse CD3 | 145-2C11 | BioLegend | 100335 |
| APC anti-mouse CD4 | RM4-4 | BioLegend | 116014 |
| FITC anti-mouse CD8a | 53-6.7 | BioLegend | 100706 |
| PE anti-mouse CD69 | H1.2F3 | eBioscience | 12-0691-83 |
