# Supplementary material for: Distinct T-cell receptor (TCR) gene segment usage and MHC-restriction between foetal and adult thymus
Source: eLife. 2024 Dec 5;13:RP93493. doi: 10.7554/eLife.93493 (PMC11620746; doi:10.7554/eLife.93493)
Supplement: Supplementary file 3. [file elife-93493-supp3.docx]

**Supplementary File 4| Primers used for TCR sequencing protocol**

| Name | Sequence | Description | Purification |
| --- | --- | --- | --- |
| TRAC3 | GAGACCGAGGATCTTTTAACTGG | RT | desalted |
| TRBC2 | GCTTTTGATGGCTCAAACAAGG | RT | desalted |
| 6N_I8.1_6N_M13_2 | [Phos]NNNNNNATCACGACNNNNNNCCAGGGTTTTCCCAGTCACGAC [SpcC3] | Ligation | HPLC |
| malphaRC1 | CAGCAGGTTCTGGGTTCTGGATG | PCR1 | desalted |
| mbetaRC1 | GGGTGGAGTCACATTTCTCAGATCC | PCR1 | desalted |
| SP2_M13 | TTC AGA CGT GTG CTC TTC CGA TCT GTC GTG ACT GGG AAAA CCC TGG | PCR1 | desalted |
| P5-SP1 | AATGATACGGCGACCACCGAGATCTACACTCTTTCCCTACACGACGCTCTTCC | PCR2 | desalted |
| mSP1-6N-I-6-aRC1 | ACACTCTTTCCCTACACGACGCTCTTCCGATCTNNNNNNGCCAATCAGCAGGTTCTGGGTTCTGGATG | SP1 | desalted |
| mSP1-6N-I-6-bRC1 | ACACTCTTTCCCTACACGACGCTCTTCCGATCTNNNNNNGCCAATGGGTGGAGTCACATTTCTCAGATCC | SP1 | desalted |
| mSP1-6N-I-7-aRC1 | ACACTCTTTCCCTACACGACGCTCTTCCGATCTNNNNNNCAGATCCAGCAGGTTCTGGGTTCTGGATG | SP1 | desalted |
| mSP1-6N-I-7-bRC1 | ACACTCTTTCCCTACACGACGCTCTTCCGATCTNNNNNNCAGATCGGGTGGAGTCACATTTCTCAGATCC | SP1 | desalted |
| mSP1-6N-I-8-aRC1 | ACACTCTTTCCCTACACGACGCTCTTCCGATCTNNNNNNACTTGACAGCAGGTTCTGGGTTCTGGATG | SP1 | desalted |
| mSP1-6N-I-8-bRC1 | ACACTCTTTCCCTACACGACGCTCTTCCGATCTNNNNNNACTTGA GGGTGGAGTCACATTTCTCAGATCC | SP1 | desalted |
| mSP1-6HN-I1-aRC1 | ACACTCTTTCCCTACACGACGCTCTTCCGATCTHNHNNHATCACGCAGCAGGTTCTGGGTTCTGGATG | SP1 | desalted |
| mSP1-6HN-I1-bRC1 | ACACTCTTTCCCTACACGACGCTCTTCCGATCTHNHNNHATCACGGGGTGGAGTCACATTTCTCAGATCC | SP1 | desalted |
| mSP1-6HN-I2-aRC1 | ACACTCTTTCCCTACACGACGCTCTTCCGATCTHNHNNHCGATGTCAGCAGGTTCTGGGTTCTGGATG | SP1 | desalted |
| mSP1-6HN-I2-bRC1 | ACACTCTTTCCCTACACGACGCTCTTCCGATCTHNHNNHCGATGTGGGTGGAGTCACATTTCTCAGATCC | SP1 | desalted |
| mSP1-6HN-I3-aRC1 | ACACTCTTTCCCTACACGACGCTCTTCCGATCTHNHNNHTTAGGCCAGCAGGTTCTGGGTTCTGGATG | SP1 | desalted |
| mSP1-6HN-I3-bRC1 | ACACTCTTTCCCTACACGACGCTCTTCCGATCTHNHNNHTTAGGCGGGTGGAGTCACATTTCTCAGATCC | SP1 | desalted |
| mSP1-6HN-I4-aRC1 | ACACTCTTTCCCTACACGACGCTCTTCCGATCTHNHNNHTGACCACAGCAGGTTCTGGGTTCTGGATG | SP1 | desalted |
| mSP1-6HN-I4-bRC1 | ACACTCTTTCCCTACACGACGCTCTTCCGATCTHNHNNHTGACCAGGGTGGAGTCACATTTCTCAGATCC | SP1 | desalted |
| mSP1-6HN-I5-aRC1 | ACACTCTTTCCCTACACGACGCTCTTCCGATCTHNHNNHACAGTGCAGCAGGTTCTGGGTTCTGGATG | SP1 | desalted |
| mSP1-6HN-I5-bRC1 | ACACTCTTTCCCTACACGACGCTCTTCCGATCTHNHNNHACAGTGGGGTGGAGTCACATTTCTCAGATCC | SP1 | desalted |
| mSP1-6HN-I6-aRC1 | ACACTCTTTCCCTACACGACGCTCTTCCGATCTHNHNNHGCCAATCAGCAGGTTCTGGGTTCTGGATG | SP1 | desalted |
| mSP1-6HN-I6-bRC1 | ACACTCTTTCCCTACACGACGCTCTTCCGATCTHNHNNHGCCAATGGGTGGAGTCACATTTCTCAGATCC | SP1 | desalted |
| mSP1-6HN-I7-aRC1 | ACACTCTTTCCCTACACGACGCTCTTCCGATCTHNHNNHCAGATCCAGCAGGTTCTGGGTTCTGGATG | SP1 | desalted |
| mSP1-6HN-I7-bRC1 | ACACTCTTTCCCTACACGACGCTCTTCCGATCTHNHNNHCAGATCGGGTGGAGTCACATTTCTCAGATCC | SP1 | desalted |
| mSP1-6HN-I8-aRC1 | ACACTCTTTCCCTACACGACGCTCTTCCGATCTHNHNNHACTTGACAGCAGGTTCTGGGTTCTGGATG | SP1 | desalted |
| mSP1-6HN-I8-bRC1 | ACACTCTTTCCCTACACGACGCTCTTCCGATCTHNHNNHACTTGAGGGTGGAGTCACATTTCTCAGATCC | SP1 | desalted |
| mSP1-6HN-I9-aRC1 | ACACTCTTTCCCTACACGACGCTCTTCCGATCTHNHNNHGATCAGCAGCAGGTTCTGGGTTCTGGATG | SP1 | desalted |
| mSP1-6HN-I9-bRC1 | ACACTCTTTCCCTACACGACGCTCTTCCGATCTHNHNNHGATCAGGGGTGGAGTCACATTTCTCAGATCC | SP1 | desalted |
| mSP1-6HN-I10-aRC1 | ACACTCTTTCCCTACACGACGCTCTTCCGATCTHNHNNHTAGCTTCAGCAGGTTCTGGGTTCTGGATG | SP1 | desalted |
| mSP1-6HN-I10-bRC1 | ACACTCTTTCCCTACACGACGCTCTTCCGATCTHNHNNHTAGCTTGGGTGGAGTCACATTTCTCAGATCC | SP1 | desalted |
| mSP1-6HN-I11-aRC1 | ACACTCTTTCCCTACACGACGCTCTTCCGATCTHNHNNHGGCTACCAGCAGGTTCTGGGTTCTGGATG | SP1 | desalted |
| mSP1-6HN-I11-bRC1 | ACACTCTTTCCCTACACGACGCTCTTCCGATCTHNHNNHGGCTACGGGTGGAGTCACATTTCTCAGATCC | SP1 | desalted |
| mSP1-6HN-I12-aRC1 | ACACTCTTTCCCTACACGACGCTCTTCCGATCTHNHNNHCTTGTACAGCAGGTTCTGGGTTCTGGATG | SP1 | desalted |
| mSP1-6HN-I12-bRC1 | ACACTCTTTCCCTACACGACGCTCTTCCGATCTHNHNNHCTTGTAGGGTGGAGTCACATTTCTCAGATCC | SP1 | desalted |
| mSP1-6HN-I13-aRC1 | ACACTCTTTCCCTACACGACGCTCTTCCGATCTHNHNNHTAGACTCAGCAGGTTCTGGGTTCTGGATG | SP1 | desalted |
| mSP1-6HN-I13-bRC1 | ACACTCTTTCCCTACACGACGCTCTTCCGATCTHNHNNHTAGACTGGGTGGAGTCACATTTCTCAGATCC | SP1 | desalted |
| P7-I8.15_SP2 | CAAGCAGAAGACGGCATACGAGATCTACCAGGGTGACTGGAGTTCAGACGTGTGCTCTTCCGATC | SP2 P7 | desalted |
| P7-I8.16_SP2 | CAAGCAGAAGACGGCATACGAGATCATGCTTAGTGACTGGAGTTCAGACGTGTGCTCTTCCGATC | SP2 P7 | desalted |
| P7-I8.17_SP2 | CAAGCAGAAGACGGCATACGAGATGCACATCTGTGACTGGAGTTCAGACGTGTGCTCTTCCGATC | SP2 P7 | desalted |
| P7-I8.18_SP2 | CAAGCAGAAGACGGCATACGAGATTGCTCGACGTGACTGGAGTTCAGACGTGTGCTCTTCCGATC | SP2 P7 | desalted |
| P7-I8.19_SP2 | CAAGCAGAAGACGGCATACGAGATAGCAATTCGTGACTGGAGTTCAGACGTGTGCTCTTCCGATC | SP2 P7 | desalted |
| P7-I8.20_SP2 | CAAGCAGAAGACGGCATACGAGATAGTTGCTTGTGACTGGAGTTCAGACGTGTGCTCTTCCGATC | SP2 P7 | desalted |
| P7-I8.21_SP2 | CAAGCAGAAGACGGCATACGAGATCCAGTTAGGTGACTGGAGTTCAGACGTGTGCTCTTCCGATC | SP2 P7 | desalted |
| P7-I8.22_SP2 | CAAGCAGAAGACGGCATACGAGATTTGAGCCTGTGACTGGAGTTCAGACGTGTGCTCTTCCGATC | SP2 P7 | desalted |
| P7-I8.23_SP2 | CAAGCAGAAGACGGCATACGAGATACCAACTGGTGACTGGAGTTCAGACGTGTGCTCTTCCGATC | SP2 P7 | desalted |
| P7-I8.24_SP2 | CAAGCAGAAGACGGCATACGAGATGGTCCAGAGTGACTGGAGTTCAGACGTGTGCTCTTCCGATC | SP2 P7 | desalted |
| P7-I8.25_SP2 | CAAGCAGAAGACGGCATACGAGATGTATAACAGTGACTGGAGTTCAGACGTGTGCTCTTCCGATC | SP2 P7 | desalted |
| P7-I8.26_SP2 | CAAGCAGAAGACGGCATACGAGATTTCGCTGAGTGACTGGAGTTCAGACGTGTGCTCTTCCGATC | SP2 P7 | desalted |
| P7_L27 | CAAGCAGAAGACGGCATACGAGAT CGCTATGT GTGACTGGAGTTCAGACGTGTGCTCTTCCGATC | SP2 P7 | desalted |
| P7_L28 | CAAGCAGAAGACGGCATACGAGAT TAAGCACA GTGACTGGAGTTCAGACGTGTGCTCTTCCGATC | SP2 P7 | desalted |
| P7_L29 | CAAGCAGAAGACGGCATACGAGAT GTAACATC GTGACTGGAGTTCAGACGTGTGCTCTTCCGATC | SP2 P7 | desalted |
| P7_L30 | CAAGCAGAAGACGGCATACGAGAT ACTAAGAC GTGACTGGAGTTCAGACGTGTGCTCTTCCGATC | SP2 P7 | desalted |
| P7_L31 | CAAGCAGAAGACGGCATACGAGAT TGTAACTC GTGACTGGAGTTCAGACGTGTGCTCTTCCGATC | SP2 P7 | desalted |
| P7_L32 | CAAGCAGAAGACGGCATACGAGAT AACAATGG GTGACTGGAGTTCAGACGTGTGCTCTTCCGATC | SP2 P7 | desalted |
| P7_L33 | CAAGCAGAAGACGGCATACGAGAT CCTTCGCA GTGACTGGAGTTCAGACGTGTGCTCTTCCGATC | SP2 P7 | desalted |
| P7_L34 | CAAGCAGAAGACGGCATACGAGAT GACCGTTG GTGACTGGAGTTCAGACGTGTGCTCTTCCGATC | SP2 P7 | desalted |
| P7_L35 | CAAGCAGAAGACGGCATACGAGAT TCTGCAAG GTGACTGGAGTTCAGACGTGTGCTCTTCCGATC | SP2 P7 | desalted |
| P7_L36 | CAAGCAGAAGACGGCATACGAGAT CACATCCT GTGACTGGAGTTCAGACGTGTGCTCTTCCGATC | SP2 P7 | desalted |
| P7_L37 | CAAGCAGAAGACGGCATACGAGAT AGGATCTA GTGACTGGAGTTCAGACGTGTGCTCTTCCGATC | SP2 P7 | desalted |
| P7_L38 | CAAGCAGAAGACGGCATACGAGAT GTCATCTA GTGACTGGAGTTCAGACGTGTGCTCTTCCGATC | SP2 P7 | desalted |
| P7_L39 | CAAGCAGAAGACGGCATACGAGAT GAACCTAG GTGACTGGAGTTCAGACGTGTGCTCTTCCGATC | SP2 P7 | desalted |
| P7_L40 | CAAGCAGAAGACGGCATACGAGAT TTACGCAC GTGACTGGAGTTCAGACGTGTGCTCTTCCGATC | SP2 P7 | desalted |
| P7_L41 | CAAGCAGAAGACGGCATACGAGAT AGGTGCGA GTGACTGGAGTTCAGACGTGTGCTCTTCCGATC | SP2 P7 | desalted |
| P7_L42 | CAAGCAGAAGACGGCATACGAGAT CATGATCG GTGACTGGAGTTCAGACGTGTGCTCTTCCGATC | SP2 P7 | desalted |
| P7_L43 | CAAGCAGAAGACGGCATACGAGAT GCCGCAAC GTGACTGGAGTTCAGACGTGTGCTCTTCCGATC | SP2 P7 | desalted |
| P7_L44 | CAAGCAGAAGACGGCATACGAGAT TTATATCT GTGACTGGAGTTCAGACGTGTGCTCTTCCGATC | SP2 P7 | desalted |
| P7_L45 | CAAGCAGAAGACGGCATACGAGAT CTGTGGCG GTGACTGGAGTTCAGACGTGTGCTCTTCCGATC | SP2 P7 | desalted |
| P7_L46 | CAAGCAGAAGACGGCATACGAGAT AACGCATT GTGACTGGAGTTCAGACGTGTGCTCTTCCGATC | SP2 P7 | desalted |
| P7_L47 | CAAGCAGAAGACGGCATACGAGAT AACTTGAC GTGACTGGAGTTCAGACGTGTGCTCTTCCGATC | SP2 P7 | desalted |
| P7_L48 | CAAGCAGAAGACGGCATACGAGAT CGCCTTCC GTGACTGGAGTTCAGACGTGTGCTCTTCCGATC | SP2 P7 | desalted |
| P7_L49 | CAAGCAGAAGACGGCATACGAGAT GACCAGGA GTGACTGGAGTTCAGACGTGTGCTCTTCCGATC | SP2 P7 | desalted |
| P7_L50 | CAAGCAGAAGACGGCATACGAGAT TCCTTGGT GTGACTGGAGTTCAGACGTGTGCTCTTCCGATC | SP2 P7 | desalted |
| P7_L51 | CAAGCAGAAGACGGCATACGAGAT AAGACACT GTGACTGGAGTTCAGACGTGTGCTCTTCCGATC | SP2 P7 | desalted |
| P7_L52 | CAAGCAGAAGACGGCATACGAGAT CTGTAATC GTGACTGGAGTTCAGACGTGTGCTCTTCCGATC | SP2 P7 | desalted |
| P7_L53 | CAAGCAGAAGACGGCATACGAGAT GAAGAAGT GTGACTGGAGTTCAGACGTGTGCTCTTCCGATC | SP2 P7 | desalted |
| P7_L54 | CAAGCAGAAGACGGCATACGAGAT TAATGAAC GTGACTGGAGTTCAGACGTGTGCTCTTCCGATC | SP2 P7 | desalted |
| P7_L55 | CAAGCAGAAGACGGCATACGAGAT TCCAGCAA GTGACTGGAGTTCAGACGTGTGCTCTTCCGATC | SP2 P7 | desalted |
| P7_L56 | CAAGCAGAAGACGGCATACGAGAT GTCCACAG GTGACTGGAGTTCAGACGTGTGCTCTTCCGATC | SP2 P7 | desalted |
| P7_L57 | CAAGCAGAAGACGGCATACGAGAT CAATAGTC GTGACTGGAGTTCAGACGTGTGCTCTTCCGATC | SP2 P7 | desalted |
| P7_L58 | CAAGCAGAAGACGGCATACGAGAT AGGTAAGG GTGACTGGAGTTCAGACGTGTGCTCTTCCGATC | SP2 P7 | desalted |
| P7_L59 | CAAGCAGAAGACGGCATACGAGAT TACTTAGC GTGACTGGAGTTCAGACGTGTGCTCTTCCGATC | SP2 P7 | desalted |
| P7_L60 | CAAGCAGAAGACGGCATACGAGAT GAAGGAAG GTGACTGGAGTTCAGACGTGTGCTCTTCCGATC | SP2 P7 | desalted |
| P7_L61 | CAAGCAGAAGACGGCATACGAGAT CATAGCGA GTGACTGGAGTTCAGACGTGTGCTCTTCCGATC | SP2 P7 | desalted |
| P7_L62 | CAAGCAGAAGACGGCATACGAGAT ATTGTCTG GTGACTGGAGTTCAGACGTGTGCTCTTCCGATC | SP2 P7 | desalted |
| P7_L63 | CAAGCAGAAGACGGCATACGAGAT CAACTCTC GTGACTGGAGTTCAGACGTGTGCTCTTCCGATC | SP2 P7 | desalted |
| P7_L64 | CAAGCAGAAGACGGCATACGAGAT ATTCTAGG GTGACTGGAGTTCAGACGTGTGCTCTTCCGATC | SP2 P7 | desalted |
| P7_L65 | CAAGCAGAAGACGGCATACGAGAT TGCTGCTG GTGACTGGAGTTCAGACGTGTGCTCTTCCGATC | SP2 P7 | desalted |
| P7_L66 | CAAGCAGAAGACGGCATACGAGAT GCCTAGCC GTGACTGGAGTTCAGACGTGTGCTCTTCCGATC | SP2 P7 | desalted |
| P7_L67 | CAAGCAGAAGACGGCATACGAGAT CCTATGCC GTGACTGGAGTTCAGACGTGTGCTCTTCCGATC | SP2 P7 | desalted |
| P7_L68 | CAAGCAGAAGACGGCATACGAGAT ATAGCGTC GTGACTGGAGTTCAGACGTGTGCTCTTCCGATC | SP2 P7 | desalted |
| P7_L69 | CAAGCAGAAGACGGCATACGAGAT TGTCGGAT GTGACTGGAGTTCAGACGTGTGCTCTTCCGATC | SP2 P7 | desalted |
| P7_L70 | CAAGCAGAAGACGGCATACGAGAT GACAGTAA GTGACTGGAGTTCAGACGTGTGCTCTTCCGATC | SP2 P7 | desalted |
| P7_L71 | CAAGCAGAAGACGGCATACGAGAT GCCGTCGA GTGACTGGAGTTCAGACGTGTGCTCTTCCGATC | SP2 P7 | desalted |
| P7_L72 | CAAGCAGAAGACGGCATACGAGAT TATCCAGG GTGACTGGAGTTCAGACGTGTGCTCTTCCGATC | SP2 P7 | desalted |
| P7_L73 | CAAGCAGAAGACGGCATACGAGAT ATTATGTT GTGACTGGAGTTCAGACGTGTGCTCTTCCGATC | SP2 P7 | desalted |
| P7_L74 | CAAGCAGAAGACGGCATACGAGAT CCAACATT GTGACTGGAGTTCAGACGTGTGCTCTTCCGATC | SP2 P7 | desalted |
| P7_L75 | CAAGCAGAAGACGGCATACGAGAT GATATCCA GTGACTGGAGTTCAGACGTGTGCTCTTCCGATC | SP2 P7 | desalted |
| P7_L76 | CAAGCAGAAGACGGCATACGAGAT TGCAAGTA GTGACTGGAGTTCAGACGTGTGCTCTTCCGATC | SP2 P7 | desalted |
| P7_L77 | CAAGCAGAAGACGGCATACGAGAT AATGTTCT GTGACTGGAGTTCAGACGTGTGCTCTTCCGATC | SP2 P7 | desalted |
| P7_L78 | CAAGCAGAAGACGGCATACGAGAT CAGCGGTA GTGACTGGAGTTCAGACGTGTGCTCTTCCGATC | SP2 P7 | desalted |
| P7_L79 | CAAGCAGAAGACGGCATACGAGAT ATTCCTCT GTGACTGGAGTTCAGACGTGTGCTCTTCCGATC | SP2 P7 | desalted |
| P7_L80 | CAAGCAGAAGACGGCATACGAGAT CTGCGGAT GTGACTGGAGTTCAGACGTGTGCTCTTCCGATC | SP2 P7 | desalted |
| P7_L81 | CAAGCAGAAGACGGCATACGAGAT GTCTGATG GTGACTGGAGTTCAGACGTGTGCTCTTCCGATC | SP2 P7 | desalted |
| P7_L82 | CAAGCAGAAGACGGCATACGAGAT TATCTGCC GTGACTGGAGTTCAGACGTGTGCTCTTCCGATC | SP2 P7 | desalted |
| P7_L83 | CAAGCAGAAGACGGCATACGAGAT ACACGATC GTGACTGGAGTTCAGACGTGTGCTCTTCCGATC | SP2 P7 | desalted |
| P7_L84 | CAAGCAGAAGACGGCATACGAGAT CCAGAGCT GTGACTGGAGTTCAGACGTGTGCTCTTCCGATC | SP2 P7 | desalted |
| P7_L85 | CAAGCAGAAGACGGCATACGAGAT GACCTAAC GTGACTGGAGTTCAGACGTGTGCTCTTCCGATC | SP2 P7 | desalted |
| P7_L86 | CAAGCAGAAGACGGCATACGAGAT TCGCCTTG GTGACTGGAGTTCAGACGTGTGCTCTTCCGATC | SP2 P7 | desalted |
| P7_L87 | CAAGCAGAAGACGGCATACGAGAT CCTACCAT GTGACTGGAGTTCAGACGTGTGCTCTTCCGATC | SP2 P7 | desalted |
| P7_L88 | CAAGCAGAAGACGGCATACGAGAT TCGCTAGA GTGACTGGAGTTCAGACGTGTGCTCTTCCGATC | SP2 P7 | desalted |
| P7_L89 | CAAGCAGAAGACGGCATACGAGAT AAGGATGT GTGACTGGAGTTCAGACGTGTGCTCTTCCGATC | SP2 P7 | desalted |
| P7_L90 | CAAGCAGAAGACGGCATACGAGAT CTAACTCG GTGACTGGAGTTCAGACGTGTGCTCTTCCGATC | SP2 P7 | desalted |
| P7_L91 | CAAGCAGAAGACGGCATACGAGAT ACAGGTAT GTGACTGGAGTTCAGACGTGTGCTCTTCCGATC | SP2 P7 | desalted |
| P7_L92 | CAAGCAGAAGACGGCATACGAGAT TCTCGGTC GTGACTGGAGTTCAGACGTGTGCTCTTCCGATC | SP2 P7 | desalted |
| P7_L93 | CAAGCAGAAGACGGCATACGAGAT ACAGTTGA GTGACTGGAGTTCAGACGTGTGCTCTTCCGATC | SP2 P7 | desalted |
| P7_L94 | CAAGCAGAAGACGGCATACGAGAT CTATGCGT GTGACTGGAGTTCAGACGTGTGCTCTTCCGATC | SP2 P7 | desalted |
| P7_L95 | CAAGCAGAAGACGGCATACGAGAT CAGGAGCC GTGACTGGAGTTCAGACGTGTGCTCTTCCGATC | SP2 P7 | desalted |
| P7_L96 | CAAGCAGAAGACGGCATACGAGAT AGGTCGCA GTGACTGGAGTTCAGACGTGTGCTCTTCCGATC | SP2 P7 | desalted |
| P7_L97 | CAAGCAGAAGACGGCATACGAGAT CAGCAAGG GTGACTGGAGTTCAGACGTGTGCTCTTCCGATC | SP2 P7 | desalted |
| P7_L98 | CAAGCAGAAGACGGCATACGAGAT ATTATCAA GTGACTGGAGTTCAGACGTGTGCTCTTCCGATC | SP2 P7 | desalted |
| P7_L99 | CAAGCAGAAGACGGCATACGAGAT TTAATCAG GTGACTGGAGTTCAGACGTGTGCTCTTCCGATC | SP2 P7 | desalted |
| P7_L100 | CAAGCAGAAGACGGCATACGAGAT CGTTACCA GTGACTGGAGTTCAGACGTGTGCTCTTCCGATC | SP2 P7 | desalted |
| P7_L101 | CAAGCAGAAGACGGCATACGAGAT AAGTAGAG GTGACTGGAGTTCAGACGTGTGCTCTTCCGATC | SP2 P7 | desalted |
| P7_L102 | CAAGCAGAAGACGGCATACGAGAT TTGAATAG GTGACTGGAGTTCAGACGTGTGCTCTTCCGATC | SP2 P7 | desalted |
| P5 | AATGATACGGCGACCACCGAGATCTACACT | QPCR | desalted |
| P7 | CAAGCAGAAGACGGCATACGAGAT | QPCR | desalted |
