# Supplementary material for: Distinct T-cell receptor (TCR) gene segment usage and MHC-restriction between foetal and adult thymus
Source: eLife. 2024 Dec 5;13:RP93493. doi: 10.7554/eLife.93493 (PMC11620746; doi:10.7554/eLife.93493)
Supplement: Supplementary file 4. [file elife-93493-supp4.docx]

**Supplementary File1f | Reagents and consumables for TCR sequencing protocol**

| Reagent | Supplier | Cat. No. |
| --- | --- | --- |
| RQ1 RNase-Free DNase | Promega | M6101 |
| RQ1 DNase 10X Reaction Buffer | Promega | M6101 |
| RQ1 Dnase Stop Solution | Promega | M6101 |
| RNase free water | Invitrogen | 10977-035 |
| dNTPs (10 mM) | Promega | U1515 |
| SuperScript III RT (200 U/µl) | Invitrogen | 18080085 |
| 5X First-Strand (FS) Buffer | Invitrogen | 18080085 |
| 0.1 M DTT | Invitrogen | 18080085 |
| RNasin | Promega | N2115 |
| Minelute PCR Purification kit | Qiagen | 28006 |
| T4 RNA Ligase Reaction Buffer | NEB | M0204L |
| Adenosine-5'-Triphosphate (ATP) | NEB | M0204L |
| PEG 8000 | NEB | M0204L |
| T4 RNA Ligase 1 (ssRNA Ligase) | NEB | M0204L |
| BSA (20 mg/mL) | NEB | B9000S |
| Hexammine cobalt(III) chloride (HCC) | Sigma | H7891-5G |
| Agencourt AMPure beads XP | Beckman Coulter | A63881 |
| 5x Phusion HF buffer | NEB | M0530L |
| Phusion Polymerase | NEB | M0530L |
| ROX Reference Dye | Invitrogen | 12223012 |
| SYBR Green I Nucleic Acid Gel Stain 10,000× | Invitrogen | S7563 |
| DMSO for molecular biology | Sigma | D8418-50ML |
| Qubit dsDNA HS Assay Kit | ThermoFisher Scientific | Q32854 |
| Qubit Assay Tubes | ThermoFisher Scientific | Q32856 |
| High Sensitivity D1000 ScreenTape | Agilent | 5067-5584 |
| High Sensitivity D1000 Reagents | Agilent | 5067-5585 |
| High Sensitivity D1000 Ladder | Agilent | 5067-5587 |
| 96-well Plates | Agilent | 5042-8502 |
| 96-well Plate Foil Seal | Agilent | 5067-5154 |
| Pippin Gel Cassette 1.5% agarose dye free 250bp-1.5kb | Sage Science | CDF1510 |
| PhiX Control V3 | Illumina | FC-110-3001 |
| MiSeq Reagent Kit v2 (500-cycles) | Illumina | MS-102-2003 |
| Eppendorf DNA LoBind Polypropylene Microcentrifuge Tube | FisherScientific | 10051232 |
| 96-well PCR plate semi skirted | Starlab | I1402-9700C |
| Adhesive PCR Plate Seal | SLS | 4ti-0500 |
| PicoPure RNA Isolation Kit | Applied Biosystems | KIT0204 |
| 500/550 Mid Output Kit v2.5 (300 Cycles) | Illumina | 20024905 |
| Blitz Away RNase spray | Serem Biotech | 40-1735-10 |
| Tris (1M), pH 7.0, RNase-free | ThermoFisher Scientific | AM9850G |
| Sodium hydroxide solution for molecular biology, 10M in H2O | Sigma | 72068-100ML |
